# Chronic Active T-Cell Mediated Kidney Rejection as a Clinically Significant Type of Allograft Loss?

**DOI:** 10.3390/diagnostics12123220

**Published:** 2022-12-19

**Authors:** Jakub Mizera, Justyna Pilch, Dorota Kamińska, Magdalena Krajewska, Piotr Donizy, Mirosław Banasik

**Affiliations:** 1 Department of Nephrology and Transplantation Medicine, Wroclaw Medical University, 50-551 Wroclaw, Poland; 2 Department of Clinical and Experimental Pathology, Division of Clinical Pathology, Wroclaw Medical University, 50-556 Wroclaw, Poland

**Keywords:** chronic active T-cell mediated rejection, kidney rejection, Banff classification, kidney transplantation, nephrology, histopathology/pathology

## Abstract

The purpose of this article is to assess the present knowledge about chronic active (CA) T-cell mediated rejection (TCMR) of a kidney. In the research authors review current Banff diagnostic criteria used in kidney rejection, focus on their possible future evolution, and investigate the role of currently available molecular methods that could be implemented into the diagnostic scheme. Research also points out previously and currently available treatment methods applied to CA TCMR and takes into account possible side effects consequent upon the therapy. Moreover, attention is being paid to the CA TCMR coincidence with other kidney rejection types such as antibody-mediated rejection (ABMR) and its influence on the treatment approach. Authors also mark the possibility of non-HLA antibodies coexistence in patients with CA TCMR and describe its possible resonance on kidney allograft function. Nonetheless, it seems that current knowledge about CA TCMR is not sufficient and requires further investigation.

## 1. Introduction

Chronic active T-cell mediated kidney rejection (CA TCMR) refers to the term that was described for the very first time during the XIII Banff Conference on Allograft Pathology in 2015 as a variant of kidney allogenic graft rejection associated with long-term graft loss [1]. Since then, the scientific community tries to establish optimal scheme of diagnostic methods and therapeutic approach. First guidelines that referred to association between inflammation of the area with interstitial fibrosis and tubular atrophy (i-IFTA) and graft failure were released officially in the Banff Classification 2015. In consequence of Banff 2015 consensus, much research was done, and a strong linkage between i-IFTA and TCMR was proved. As a result, at the Banff Classification 2017, the diagnostic criteria for CA TCMR were revised, including i-IFTA as an elementary lesion [2]. The latest updates from 2019 Banff Classification finally enabled diagnosing CA TCMR [3]. Even though a lot of effort is dedicated to solving this problem, the guidelines regarding diagnostic criteria and therapy remain questionable and not efficiently described, which yields lack of effective treatment. This research is focused on comparison of nowadays diagnostic criteria appliance in patients after kidney transplantation (KT) and the influence that it has on making prognosis regarding graft rejection and choosing therapeutic approach.

## 2. Materials and Methods

A comprehensive analysis of literature was conducted including articles and journals published up to September 2022. The PubMed accessed on 31 September 2022, and Google Scholar accessed on 31 September 2022 databases were searched for variations of keywords: chronic active T-cell mediated rejection, kidney rejection, Banff classification, immunosuppressive treatment, kidney transplant rejection, allograft loss, antibody mediated rejection, molecular diagnostics of kidney rejection, kidney rejection treatment, immunosuppression. Moreover, the PubMed Advanced Search Builder was used to search for the following phrases: (CA TCMR) OR (chronic active rejection) OR (ABMR) OR (antibody mediated rejection) AND (Banff) AND (kidney rejection) OR (allograft loss) and others. The literature search was conducted by 2 authors and about 9700 were filtered. We included 41 papers which specifically concerned the problem of kidney allograft rejection. Abstracts in languages other than English were excluded.

## 3. Characteristic Feature of Chronic Active T-Cell Mediated Rejection in Transplant Biopsy and Current Diagnostic Criteria

### 3.1. Overall View on Parameters Used in the Renal Histopathological Banff Classification

The Banff system used for classification of renal histopathological changes focuses mostly on diagnostic features present in graft rejection. It comprises of 6 categories where CA TCMR was classified in Category 4 which refers to histopathological changes caused by T-cells [4].

There are various histopathologic parameters included in the Banff classification. The most important selected parameters which should be included in the histopathologic reports of kidney transplant biopsies were summarized below (Table 1) [3].

Moreover, depending on the grade of CA TCMR the following histological changes may be recognized:

1. The Banff lesion score t (tubulitis)—assesses the degree of inflammatory process within non-scaring cortical tubules’ epithelium. According to the definition of tubulitis, it is an inflammation located in the basolateral part of renal tubule epithelium caused by the presence of mononuclear cells. The score is measured in longitudinally cut tubules and defined by the quantity of mononuclear cells per 10 epithelial cells of the tubules. Tubulitis in CA TCMR refers to grades IA and IB. Lesions are considered in cortical tubules in non-scoring interstitial areas except ones with severe atrophy. Severely atrophic tubules are specified by the diameter lower than 25% of non-affected tubules.
2. The Banff lesion score v (intimal arteritis)—this score refers to intimal arteritis, which is the presence of leukocytes, mainly lymphocytes, in the subendothelial space of at least one artery. Lesions penetrating the intima deeper are graded as stage II of CA TCMR.
3. The Banff lesion score cv (vascular fibrous intimal thickening)—this term concerns the most severely affected artery in the specimen and assesses the thickening of arterial intima. These lesions are presented in grade II of CA TCMR.
4. The Banff lesion score ti (total inflammation)—this score assesses the intensity of total cortical inflammation. If the score is at least 2 together with i-IFTA score the diagnosis of CA TCMR grade IA or IB can be considered [3].
5. The Banff lesion score t-IFTA (tubulitis in areas of interstitial fibrosis)—this score has been introduced to differ the location of inflamed tubules. It refers to tubules located within scared cortex and does not include these ones present in preserved cortex. It has been proved that there is no effect of isolated t score on graft outcome—so the need of differentiation of location of inflamed tubules has been noticed. t-IFTA may be observed in CA TCMR as a moderate lesion in stage IA and as a severe lesion in stage IB [5].
6. The Banff lesion score i-IFTA (inflammation in area of IFTA)—this criterion is important in recognition of CA TCMR. Without the presence of this type of lesions the diagnosis of CA TCMR cannot be made. This score refers to the inflammatory process in the scarred cortex. As it is mentioned above if the score is at least 2 together with ti score the diagnosis of CA TCMR grade IA or IB can be considered [3].

### 3.2. Current CA TCMR Diagnostic Criteria

There are 2 points that need to be highlighted regarding CA TCMR. Even though inflammation in areas of the cortex with interstitial fibrosis and tubular atrophy (i-IFTA) is an elementary lesion of CA TCMR, it is not sufficient to make this diagnosis. It requires parallel presence of moderate tubulitis involving cortical tubules different than severely atrophic tubules and at least a moderate degree (grade 2) of total cortical inflammation. Moreover i-IFTA cannot be regarded as specific lesion for CA TCMR. It can be seen in various types of tissue injury including ABMR or BK virus nephropathy. In opposition to previous criteria, it has been suggested that for clarity and future investigation tubulitis should be independently scored in areas of cortical IFTA (t-IFTA score) and in areas of preserved cortex (t score). It may be useful for clinicians to assess the impact of these different forms of tubulitis on treatment response and graft survival [6].

At Banff 2019 Conference updated criteria were released and CA TCMR was staged into 3 grades: IA, IB and II: [3].

- Grade IA: interstitial inflammation which involves >25% of the total cortex (ti2 or ti3) and >25% of scarred cortex (i-IFTA2 or i-IFTA3) with moderate tubulitis (t2 or t-IFTA2) involving at least one tubule but without including severely atrophic ones at the same time;
- Grade IB: interstitial inflammation which involves >25% of the total cortex (ti2 or ti3) and >25% of scarred cortex (i-IFTA2 or i-IFTA3) with severe tubulitis (t3 or t-IFTA3) involving at least one tubule but without including severely atrophic ones at the same time;
- Grade II: chronic allograft arteriopathy as in previous criteria.

It’s crucial to keep in mind that lesions characteristic for Grade II CA TCMR may also occur as evidenced of chronic active or chronic ABMR or ABMR mixed with TCMR.

### 3.3. Molecular Diagnostics of CA TCMR

Clinical and histological criteria, used so far in aim to assess kidney allografts, are not sufficient in reflecting a complex pathogenesis of graft failure. Their greatest limitations are the descriptive and semi-quantitative character of diagnostic assessments, which yield in lack of knowledge about the actual patomechanisms behind kidney graft rejection [7]. The answer to this issue is the implementation of molecular techniques into a diagnostic approach, hence this topic was one of the main themes during the XV Banff Conference held in September 2019. Moreover, the Banff Molecular Diagnostic Working Group (MDWG) updated other participants about the progress achieved in this field and outlined a further plan for the development of the role of molecular diagnostics in solid organ transplantation [8].

MDWG also reported the construction of a multiorgan transplant gene panel, called the Human Organ Transplant (B-HOT) panel. The final B-HOT panel consists of 758 genes covering the most important genes from the core pathways and processes which relate to host responses to rejection of transplanted organ or tissue, drug-induced toxicity, tolerance, transplantation-associated viral infections and 12 internal reference genes used for quality control and normalization. Genes considered as kidney specific are: AQP2, KAAG1, NPHS1, NPHS2, SLC12A3 and UMOD. The panel is available commercially and believed to smooth the path of multicenter collaborative clinical research by leveraging archival samples and allowing the enlargement of an open-source database of standardized analyses. The creators of B-HOT trust that a pathway and pathogenesis based molecular approach will be of value to researchers and promote clinical trials and therapeutic decision-making. Prospective studies proved a strong association between molecular transcript patterns and histological Banff lesions. Strong linkage with the diagnosis was also revealed. Still, some discrepancies were also identified so it is crucial to perform further investigations to reveal the optimal integration of molecular and histology biopsy features [8].

Other methods used in molecular diagnostics:

- Reverse transcription polymerase chain reaction (RT-PCR) was the first molecular method ever used in studies regarding transplant rejection. It allows the evaluation of transcripts associated with cytokine burden or T-cell. Research based on this method enabled to establish associations of:
  - acute graft rejection with higher mRNA expressions of several cytkines [9] and Toll-like receptors [10];
  - chronic graft injury with profibrogenic cytokines and chemokines [11];
  - therapy-resistant rejection with Fas ligand gene expression [12];
  - inferior outcome of early acute rejection with lower expression of regulatory transcript FOXP3 and CD20 [8].
- Microarray technology enables large-scale analysis of transcriptomic data. Molecular tests for TCMR based on microarray analysis of mRNA expression provide a histologically independent measurement against which the relative validity of new algorithms for histologic diagnosis of TCMR can be determined. Molecular TCMR scores reflect the interaction of relevant effector T cells and antigen-presenting cells in the interstitium and are strongly correlated with histological lesions and diagnosis of TCMR. Studies showed the feasibility and utility of central microarray-based molecular measurements to assess disease states in transplant biopsies and demonstrate the possibility of molecular testing of biopsies combined with histology to improve our understanding of these diseases [13,14].
- Transcriptomic profiling of kidney graft biopsies which results demonstrate higher specificity in comparison to semi-quantitative histology assessment. Moreover, a smaller amount of tissue samples is required for adequate assessment [13].

Taking into consideration the assumption about greater ability of transcripts to reflect ongoing processes in kidney transplant, Halloran et al. implemented a definition for pathogenesis-based transcript sets (PBTs). Its validity and importance were confirmed later on by different research groups, including studies of Mueller at all and Sis et al. [15,16]. Transcriptomic data is expected to improve prognosis and diagnostic approach in kidney transplant settings.

- The NanoString nCounter system based on Formalin-Fixed Paraffin-Embedded (FFPE) biopsy is a modern, practical technology. It identifies similar associations of transcript with the histologic and molecular phenotypes as those reported in microarray studies. The NanoString system provide comparable results between fresh frozen samples and FFPE, with a higher sensitivity than that of microarrays and almost equal to reverse transcription polymerase chain reaction (RT-PCR) without a need of enzymes and requires only a single reaction per one sample despite the level of multiplexing. The system is approved for clinical use and introduced in pathology laboratories, equipped with analytical software and an automated platform being easily operated by lab technicians.
- NGS (next generation sequencing) is a relatively new technology based on DNA sequencing that has revolutionized genomic research [17]. It could be possibly useful to analyze transplant failure resulting from organ rejection, which may originate from an exaggerated or/and aberrant immune response. Basing on a subset of studies [18], Tsai-Hung et al. presumed that sequencing the expressed genes of B- and T-cells in the immune repertoire (iR) could provide clinical implications in the management and prediction of renal transplant rejection caused by immune diversity. Using NGS, they conducted the analysis in which they monitored the sequence change of CDR3 (complementary determining region 3) in BCR (B-cell receptors) IGH (immunoglobulin heavy-chain) iR in patients after kidney transplantation. The results of this research led to the conclusion that immune diversity is closely related to graft loss. However, they underlined the issue of an insufficient number of samples. Hence, interpreting an individual’s immune response regarding the iR information undoubtedly deserves further investigation [18].

The majority of published studies, in which both molecular diagnostic methods and histological diagnostic criteria were used, proved strong association of transcript patterns with histological lesions classified in Banff. Both of these methods consequently led to similar or the same diagnosis; however, also discrepancies between them have been identified. Further research should be provided in the aim to unite these both approaches and elaborate an optimal diagnostic scheme of potential risk of graft failure and improve outcome of kidney transplantation [8].

### 3.4. Evaluation of Current Diagnostic Criteria Based on Recent Clinical Experience

First report on the prevalence and prognosis of CA TCMR in clinical practice was published by Nakagawa et al. in 2020 [19]. In this clinical research authors assessed the significance and accuracy of diagnostic criteria provided during the Banff 2017 Conference.

They remarked on the need to separate the analysis of t ≥ 2 and ti ≥ 2 lesions in patients who were included into i-IFTA ≥ 2 group because of the lack of interaction of the composite graft endpoint between these 2 lesions. Moreover, they observed that t ≥ 2 group in comparison to t < 2 group had worse prognosis. when the assessment was made in non-scarred areas. However, this observation may not be applied to scarred areas, where the difference in graft prognosis was not noticed. Also, no difference in prognosis between ti ≥ 2 and ti < 2 groups was proved.

Nonetheless, the authors found Banff 2017 criteria effective in identifying patients with unfavorable prognosis among patients for whom allograft function appears to be stable. The authors suggested a need to verify the validity of the combination of the ti lesion and i-IFTA lesion [19].

According to clinical observations, the clarification of diagnostic criteria of CA TCMR was a main purpose of XV Banff Conference which took place in 2019 [3]. Substantively, changes were provided into Banff 2019 Classification, in which grade I of CA TCMR was divided into grade IA and IB based on the severity of tubulitis (t score) [3]. To sum up, in the working definition of CA TCMR three histological scores: i-IFTA, t and ti play the main role.

The most recent research regarding the assessment of Banff 2019 diagnostic criteria was held by Helgeson et al. [6] who studied the histological changes in biopsies of 2 Deterioration of Kidney Allograft Function (DeaKAF) cohorts. The research led to important conclusions, from which the first one claims that a threshold for diagnoses including i-IFTA, ti and t should be reconsidered due to similar death-censored graft survival (DC-GS) in biopsies with i-IFTA = 1 and i-IFTA ≥ 2 with simultaneous ti ≥ 2 and t ≥ 2. Also, the observation in the prospective cohort has shown that DC-GS was significantly worse when associated with i-IFTA = 1 in comparison to i-IFTA = 0.

Secondly, they questioned the accuracy of diagnostic category CA TCMR itself, due to the fact that scores i-IFTA, ti and t all concomitantly ≥2 can be recognized not only in the previous presence of TCMR but also in case of the absence of preceding TCMR. Even though observations in 1-year protocol biopsies support the diagnostic criteria for CA TCMR established during XV Banff Conference, the study of Halloran et al. [20] based on late indication biopsies, queried the validity of current CA TCMR definition which implies that the only immunologic cause of decreased graft survival in such diagnosis is TCMR. Similar conclusion was made also in the recent study on two cohorts (prospective and C-S). Those two studies assert that especially in late indication biopsies, i-IFTA was more likely to be a result of ABMR than of TCMR. Moreover, in some cases i-IFTA was preceded by neither TCMR nor ABMR.

The data obtained during the research also support the hypothesis that late graft failure is claimed to be a result of a new active process rather than previous injury. It was confirmed that i-IFTA is related to underimmunosuppression which is associated with the development of ABMR and donor specific antibody (DSA). The last deduction from the study indicates that i-IFTA is significantly associated not only with tubulitis but also with other scores like g, ptc, DSA and C4d [3,21].

### 3.5. The Future Prospect for Changes in Diagnostic Criteria

All of those discoveries challenge the current CA TCMR definition. First of all, the revision of terminology should be considered. Term “CA TCMR” indicates that the process is chronic, but it actually comprises three histologic scores associated with acute instead of chronic change (i-IFTA, t, ti). According to Helgeson et al., the new definition should designate an acute process combined with non-specific chronic changes. Moreover, Loupy et al. [22], based on previously provided research, reported a need of revisiting CA TCMR grade I category as a part of chronic changes but without automatically assuming TCMR as its only possible cause.

Clinicians also point out that the Banff diagnostic criteria should be applicable not only for protocol biopsies but also for indication ones at any time after transplantation [6].

Arrangements made so far indicate the need to analyze the combination of concurrent scores present in diagnosis of TCMR and revision of threshold of i-IFTA. This issue is being addressed by Nickeleit and Randhawa who are the leaders of TCMR working group, aiming to integrate i-IFTA into classification, reevaluate thresholds for t and i and add other findings to diagnostic criteria [3].

## 4. Treatment Approach

### 4.1. Assessment of Efficiency of Current and Previous Treatment Methods

Kidney transplantation (KT) is the best method for kidney replacement therapy (KRT) because of high patient survival rates and quality of life (QoL) [23]. Nonetheless this treatment requires immunosuppressive therapy and currently the occurrence of CA TCMR is being associated by the scientists with insufficient immunosuppression [24]. As mentioned before, it is linked with the presence of characteristic lesions such as i-IFTA which suggests that immunosuppressive therapy could be beneficial for patients as a treatment method [25]. Nonetheless the preferential treatment method hasn’t been established yet and various clinical studies are being performed.

Basing on recent research, the medications which can be potentially helpful in CA TCMR treatment are methylprednisolone (MP, 4 mg/d), tacrolimus (Tac, minimal concentration 5–8 ng/mL), mycophenolate mofetil (MMF, 500–1500 mg/d), basiliximab (20 mg in days 0 and 4), everolimus (EVR, minimal concentration 3–8 ng/mL) and anti-thymocyte globulin (ATG). According to clinical case reports combining doses of maintenance oral immunosuppressive agents such as Tac, MMF, MP and EVR with ATG administration together with steroid pulse therapy may give promiscuous results. In the outcome of the treatment in some cases part of lesions characteristic for CA TCMR subsided and in none of 3 patients a deterioration of eGFR was observed [26].

Different studies have shown that only in a small subset of cases the improvement in kidney function in patients suffering from CA TCMR can be achieved with immunosuppression. Treatment provided in that research consisted of 500 mg i.v. of methylprednisolone for 3 days followed by oral administration of prednisone 5 mg daily dose for over 4 weeks. In addition, if the stage of CA TCMR was IB, the treatment was combined with ATG (1.5 mg/kg daily for 4 days). Only 20% of patients achieved at least 50% eGFR recovery at 4 weeks after biopsy. The association between the stage of CA TCMR and treatment response was noticed. Patients with grade IB tended to have a lower response than the ones with IA grade of CA TCMR. In addition, the observed response rate was better in patients who didn’t meet criteria for co-existing acute TCMR what suggests that treatment response is not linked with the treatment of acute TCMR. No significant difference was noticed between patients with severe, moderate, or mild parenchymal scarring in biopsies. Nonetheless, increased immunosuppressive therapy may be contraindicated in some cases where criteria for acute TCMR are not met and severe parenchymal scaring is recognized [25].

Studies presented above were performed on a modest group of patients and as a result of this fact, discovered findings cannot be conclusive for establishing criteria for treatment of CA TCMR. Yet, achieved results seem to be inquisitive and need further investigation.

### 4.2. Immunosuppression in Treatment of CA TCMR

Some clinicians hesitate about the treatment of CA TCMR because of the possible risks of immunosuppressive therapy as it is mentioned in Section 4.3 below. One of the possible medication groups to prevent graft from rejection are calcineurin inhibitors such as tacrolimus, which are characterized by excellent short-term therapeutic results. However, the chronic nephrotoxicity of these medications is the major disadvantage of present immunosuppressive regimens. A significant relationship between the concentration and the toxicity or possible rejection was noticed. Ranges noted in the research are very broad from 5 to 25 ng/mL, depending also on the time after transplantation. All those observations possibly suggest that these medications have a very narrow therapeutic window and doses should be differently chosen depending on each patient’s various conditions [27]. In another research the association was observed between graft tacrolimus concentration and nephrotoxicity (828 pg/mg tissue) [28].

Factors which contribute to its side effects include tacrolimus’ systemic levels; exposure to metabolites of tacrolimus, local renal exposure to tacrolimus; local vulnerability factors for calcineurin inhibitors (CNI) nephrotoxicity such as age of a kidney, local hepatic, and intestinal cytochrome P450A3, local renal P-glycoprotein and renin-angiotensin-aldosterone (RAA) system activation. Potentially nonreversible nephrotoxicity of tacrolimus may lead to kidney graft loss and its diagnosis should be made after exclusion of any different causes of allograft dysfunction and also with reference to the clinical context [29].

There are available other immunosuppressive treatments such as MMF which is deprived of nephrotoxicity. Its mechanism of action is based on depletion of guanosine nucleotides in T and B lymphocytes and also the inhibition of their proliferation. As a result, MMF suppresses not only cell-mediated immune response but also antibody formation. Because of this mechanism, MMF decreases both acute and chronic rejection in graft recipients and likewise can be used in some other nephropathies [30].

Another medication which can be possibly used in the treatment of CA TCMR is ATG. It is administered mostly for steroid-resistant TCMR. However, basing on small clinical study it is encouraged for the prompt usage upon CA TCMR diagnosis [26]. On the other hand, the high probability of undesirable events including neutropenia and Cytomegalovirus (CMV) antigenemia often results in the stoppage of escalated immunosuppression. The conclusion is that ATG administration often needs CMV prophylaxis with the use of Valganciclovir (VGCV) [31]. It suggests that every patient, especially ones with high risk of infection and in danger of neutropenia, requires a customized treatment strategy.

### 4.3. The Revision of the Need of CA TCMR Treatment

After the Banff 2017 conference where CA TCMR criteria were revised the study was performed to assess if CA TCMR is diagnosed and treated worldwide. It has proven that CA TCMR was diagnosed in more than 90% of cooperating clinical centers and treated with steroids or other immunosuppressive medications in more than 80% of cases. Precisely 36% of cases were treated routinely, 49% under condition and 15% rarely. Those clinicians who treated CA TCMR under condition depended the therapy on the score i and v in the Banff lesion score (49%), on the lack of severe IFTA (47%) or on the cause of biopsy (4%). Doctors who hesitated to provide the treatment referred to the lack of clinical data (64%), some believed that risk outweighs benefits or pointed out the experience of ineffectiveness (27%). In addition, in 9% of cases, pathologists did not diagnose CA TCMR at all.

Patients were treated mostly with steroids (25%), 18% with steroids combined with ATG if the grade of illness was diagnosed as IB, 17% with increased baseline immunosuppressants and 16% with ATG if there was no response for steroids treatment [3]. It is observed that treatment of CA TCMR/i-IFTA can improve kidney function. Based on these observations i-IFTA cannot be considered as an irreversible nonspecific lesion of the tissue. Instead, it should be seen as an ongoing, active tissue injury susceptible to treatment in the proper context of the underlying inflammatory process [32].

There is a possible relationship between CA TCMR and preceding acute TCMR, mixed ABMR with acute TCMR or active AMBR. It was examined that 55% of treated CA TCMR matched those criteria. The share of responders was not suggestively different between patients with and without prior episodes of rejection. It was examined that 30% of treated recipients had donor-specific antibodies even though they didn’t meet the Banff 2017 criteria for ABMR. It seems that the association to prior rejection is a complex phenomenon in an evaluation of biopsies from the kidney allograft function studies cohort. Although former rejection was more often observed in i-IFTA, the majority of CA TCMR biopsies did not have preceding acute rejection and no type of rejection was more common than others [6].

It is crucial to compare acute TCMR, i-IFTA attributable to other diseases like ABMR or recurrent glomerulonephritis and IFTA without inflammatory process to assess if immunosuppressive treatment of CA TCMR is related to its T cell mediated immunologic pathomechanism or if it represents nonspecific anti-inflammatory mechanism on any form of i-IFTA. It could potentially be advantageous in assessment of determination of immune mediated mechanisms of injury which can be treated by immunosuppressive therapies versus mechanisms which are not immunological mediated which seem to be more resistant to therapy [32].

Current findings put the spotlight on new treatment methods including the application of nanoparticles in patients with kidney grafts or other kidney diseases. The use of nanoparticles may reduce and prevent ischemic reperfusion injury, more efficiently deliver the drug to the transplant site while avoiding systemic effects and also accurately identify and visualize the affected area. By making it possible, it has the potential to revolutionize kidney transplantation [33].

Although some clinical research were performed and results are promiscuous, there is still a need for big, randomized research where patients would be treated with various immunosuppressive agents and combinations of those medications to work out an optimal scheme of CA TCMR treatment.

## 5. The Impact of ABMR Coincidence on the CA TCMR Prognosis

### 5.1. Significance of Non-HLA Antibodies in Patients with TCMR

The most important immunological barrier in organ transplantation is the phenomenon of recognition of non-self antigens on donor cells by the recipient’s immune system. The human leukocyte antigens (HLA) provided in the major histocompatibility complex (MHC) are seen as the most important alloantigens in transplantation. Differences between donor and recipient cells cannot only be seen in MHC. Other cause of donor’s immunological response are minor histocompatibility antigens (mHA) which consist of all mismatched proteins that are able to introduce directed immune response because of its sufficient antigenicity [34]. It was observed that decreased long term graft survival is linked with the formation of post-transplant antibodies against non-HLA autoantigens. Moreover, recent studies compelling clinical and experimental findings suggest that non-HLA antibodies directed against autoantigens may contribute to both acute and chronic ABMR [35]. Moreover, the presence of non-HLA antibodies does not only refer to kidney graft loss but has also been found in other transplant rejection types such as hand allografts rejection [36]. T cells play a very important role in the formation of non-HLA antibodies. Current understanding of alloimmunity points out 2 main ways of allorecognition: direct and indirect one. Direct allorecognition is connected with the phenomenon of introduction of donor’s Antigen-presenting cells (APCs) together with the transplant to recipients body. It is proved that donor’s APCs interact with recipient T cells resulting in strong immune response. This way of allorecognition is especially important for acute TCMR. Yet, it is not entirely understood whether the non-self HLA molecule serves itself as the ligand for recipient’s alloreactive T-cells or if a different peptide range, presented in the area of peptide-binding groove of the non-self HLA molecule is crucial. On the other hand, indirect allorecognition is based on the recognition of allopeptides presented on MHC class II by professional APCs such as dendritic cells or monocytes by T-cell receptors (TCRs). Thus, indirect allorecognition does not only include specific peptide fragments from alloreactive HLA molecules but generally all mismatched and polymorphic proteins. The process of recognition of peptides shed from the transplanted organ is likely the same as the way of internalization of exogenous antigens by recipients APCs before being presented in self-MHC class II molecules to CD4+ cells [34].

Gareau et al. performed a study to assess possible pathogenic effects of non-HLA antibodies on kidney graft survival and discovered that Ab against the angiotensin II type 1 receptor AT1R-Ab may contribute to higher risk of allograft rejection. Moreover, the role of other non-HLA antibodies in rejection was examined in this study. It has been tested in a cohort of 101 patients pre- and post- transplant to see if there was a presence of any non-HLA antibodies, in particular AT1R-Ab. It has been revealed that individuals with positive pre-transplant AT1R-Ab were at the higher risk of development of de novo donor-specific Ab (dnDSA) in contrast to patients negative for AT1R-Ab. In addition, it has been noticed that pre-transplant positivity for AT1R-Ab was connected with the occurrence of TCMR in the first year after transplant. Nonetheless it did not predict allograft loss independent of de novo donor-specific antibodies (dnDSA).

Moreover, the study has shown a significant association between the AT1R-Ab positivity and positivity for Ab against the endothelin A type 1 receptor (ETAR-Ab) [37]. Researchers pointed out the need to perform a prospective study regarding high prevalence of AT1R-Ab pre-transplant and its association with TCMR and possible treatment methods in such patients. It ought to determine if AT1R blockade or more intense immunosuppression can possibly have a positive outcome in these patients [38,39].

It is recommended to perform protocol biopsy routinely at the initial detection of dnDSA and within the first year with preexisting DSA. In those patients with positive biopsies, it is advised to maximize baseline immunosuppression, any associated TCMR should be treated, and adherence stressed. However, it is still questioned if antibody-reduction treatment ought to be initiated. It is considered that testing blood for a donor’s DNA or less invasive gene profiling methods may play a role in follow-ups in patients with negative initial biopsies.

However, if a protocol biopsy is positive but the detectable HLA-DSA is absent it should be determined whether non-HLA-DSA ought to be examined for either in particular or on a genome-wide basis. It is also important to find out the way of the best treatment for these patients [40].

### 5.2. The Influence of ABMR on Treatment Methods

The production of alloantibodies refers to almost 45% of patients after KT. The development of DSAs in more than half of the patients was associated with rejection episodes. Patients with anti-donor alloreactivity showed worse renal function [41]. Thus, it seems that patients diagnosed with CA TCMR and AMBR together may constitute a significant group of patients.

The coexistence of ABMR is not without significance for the management of CA TCMR. A study by Parkes et al. [42] suggests that to a certain point both TCMR and ABMR engage in their pathomechanisms the same effector process leading to graft rejection.

Authors of the study claim that a potential source of variability in the clinical pictures between TCMR and ABMR is the different localization of the effector cells, which are, respectively, NK cells in AMBR and effector T cells in TCMR. This knowledge could lead to the conclusion that both types of transplant rejection can be treated with the same agents with similar effectiveness, but this hypothesis requires further research.

Research suggests that activation of both mechanisms depends on calcineurin, which indicates that calcineurin inhibitors should be effective both in ABMR and TCMR treatment. The major problem of this therapeutic approach is the need to limit the doses of calcineurin inhibitors due to their toxicity; however, acceptable therapeutic doses are at sub-saturation levels, unable to completely block calcineurin.

Other agents considered by authors as potentially effective in both TCMR and ABMR are anti-thymocyte globulin, alemtuzumab and anti-CD2. The effect of these medications is the depletion of both T cells and NK cells and their use in graft rejection treatment should also be further explored [42].

Moreover, in the survey conducted by Sood et al. [43] in the USA among research centers dealing with the treatment of kidney allograft rejection, the most frequently reported treatment model for CD4+ ABMR was combination of PP/IVIG/rituximab (67%). Moreover, in the case of CD4+ ABMR, bortezomib was considered more often than in CD4− AMBR (25% vs. 16%).

## 6. Significance of CA TCMR for Transplant Survival

In spite of advances in post-transplant management approximately 40 percent of grafts don’t survive in the recipient’s organism more than 10 years after the transplantation. CA TCMR is a one of two principal types of chronic kidney transplant rejection (CKTR) is a progressive allogenic immune process, often clinically silent yet progressive which leads to deterioration of graft function and its cumulative injury. The process of CKTR is often manifested at 1 year after the transplantation and it is characterized by decreased graft function often accompanied by proteinuria and hypertension [24].

Nakagawa et al. Performed a study to assess the scale of rejection in patients suffering from CA TCMR. Research performed on a group of 406 patients has shown that patients with CA TCMR or AMBR had worse estimated graft survival than ones with normal or acute TCMR. It also revealed that there was no difference in graft survival between patients with different grades (I and II) of TCMR. Performed analysis proved that individuals with CA TCMR were more likely to develop allograft dysfunction and its loss compared with normal tissue also after adjusting by significant clinical factors [28].

The prognosis of CA TCMR and graft survival largely depends on the reversibility and severity at the time of making a diagnosis. Yet, it is still challenging to diagnose early changes before the graft is irreversibly damaged. Also, foregoing TCMR is thought to be correlated with the development of CA AMBR dnDSA. Due to its complex pathogenesis, lack of clinical data and the irreversibility at the time of diagnosis the management of CKTR is still an exacting task. Nonetheless some studies about promising approach methods are being held right now. In the very near future new technologies such as AI-based assistance, computational biology and single cell genomics may be identified and implemented in clinical practice for the therapy of CKTR [24].

## 7. Conclusions

This study aimed to investigate the relevance of currently used Banff diagnostic criteria and led to conclusions that with the development of those criteria relating to different types of renal allograft rejection and especially to CA TCMR, the number of patients in whom renal rejection can be diagnosed at an early stage has increased significantly comparing to the past when physicians were unable to spot the often silent and early stages of various types of rejection. Nonetheless, researchers are aware of the need for further studies and changes implemented into diagnostic criteria in order to constantly increase graft survival.

Despite the huge progress in establishing an ideal diagnostic scheme, an optimal treatment regimen has not yet been set up, so it seems crucial to find a target and universal treatment algorithm which could possibly be applied in every transplant unit.

This review indicates the need to reconsider holistically the problem of CA TCMR. Perhaps also a comprehensive analysis that takes into account the likelihood of coexistence of other types of rejection, such as ABMR, could improve treatment regimens and increase graft survival in patients.

## Figures and Tables

**Table 1:** Summary of Banff lesion score classification.

Banff Lesion Score	Abbreviation	0	1	2	3
Inflammation in non-scarred cortex	i	Absent/minimal (Less than 10% of non-scarred cortex inflamed)	Mild, (10–25% of non-scarred cortex inflamed)	Moderate, (26–50% of non-scarred cortex inflamed)	Severe, (more than 50% of non-scarred cortex inflamed)
Tubulitis	t	None	Mild, (1–4 mononuclear leukocytes per tubular cross-section or 10 tubular epithelial cells in most severely involved tubule)	Moderate, (5–10 mononuclear leukocytes per tubular cross-section)	Severe, (more than 10 mononuclear leucocytes per tubular cross-section)
Intimal arteritis	v	None	Mild (at least 1 leukocyte directly under the endothelium of at least 1 artery)	Moderate, (as grade 1, but with ≥25% luminal occlusion)	Severe, (with arterial fibrinoid necrosis or transmural inflammation)
Glomerulitis	g	None	Mild, (with at least 1 leukocyte AND associated endothelial swelling occluding more than 50% of at least 1 capillary lumina in at least 1 but less than 25% of glomeruli)	Moderate, (as grade 1 but involving 25–75% of glomeruli)	Severe, (as grade 1 but involving more than 75% of glomeruli)
Peritubular capillaritis	ptc	Minimal, (with less than 3 leukocytes in the most severely involved cortical PTC and/or leukocytes in less than 10% of cortical PTCs)	Mild, (with at least 1 leukocyte in at least 10% of cortical PTCs AND 3–4 leukocytes in the most severely involved PTC)	Moderate, (as grade 1 but with 5–10 leukocytes in most severely involved PTC)	Severe, (as grades 1–2 but with more than 10 leukocytes in most severely involved cortical PTC)
Interstitial fibrosis in cortex	ci	Minimal, (not more than 5% of fibrosis)	Mild, (6–25%)	Moderate, (26–50%)	Severe, (more tahn 50%)
Tubular atrophy in cortex	ct	None	Mild, (1–25% of atrophy)	Moderate, (26–50%)	Severe, (more than 50%)
Arterial intimal fibrosis	cv	None	Mild, (present but with not more than 25% narrowing of luminal area in the most involved artery)	Moderate, (26–50% luminal narrowing)	Severe, (more than 50% luminal narrowing)
Chronic glomerulopathy	cg	None	1a (early mild, no GBM double contours by LM but subendothelial neo-densa in at least 3 glomerular capillaries by EM with associated endothelial cell enlargement and/or subendothelial electron-lucent widening, 1b (mild, GBM double contours by LM in 1–25% of glomerular capillaries by LM in the most severely involved glomerulus)	Moderate, (double contours by LM in 26–50% of capillaries)	Severe, (double contours by LM in more than 50% of capillaries)
Total cortical inflammation	ti	Absent/Minimal, (less than 10% of inflamed cortex)	Mild, (10–25%)	Moderate, (26–50%)	Severe, (more than 50%)
Inflammation in scarred cortex	i-IFTA	Absent/Minimal, (less than 10% of non-scarred cortex inflamed OR if the extent of cortical IFTA is less than 10%)	Mild, (10–25% of scarred cortex inflamed)	Moderate (26–50% of scarred cortex inflamed)	Severe, (more than 50% of scarred cortex inflamed)
Tubulitis in tubules within scarred cortex	t-IFTA	None	Mild, (1–4 mononuclear leukocytes per tubular cross-section or 10 tubular epithelial cells in most severely involved tubule)	Moderate, (5–10 mononuclear leukocytes),	Severe, (more than 10 mononuclear leukocytes)
Intrarenal polyomavirus load level	pvl	None	Mild, (virus positive cells in not more than 1% of tubules)	Moderate, (more than 1% and less than 10%)	Severe, (at least 10%)

## Data Availability

Not applicable.

## Abbreviations

The following abbreviations are used in this manuscript:

CA	chronic active
TCMR	T-cell mediated rejection
ABMR	antibody mediated rejection
i-IFTA	inflammation of the area with interstitial fibrosis
KT	kidney transplantation
t-IFTA	tubulitis in areas of interstitial fibrosis and tubular atrophy
MDWG	Molecular Diagnostic Working Group
B-HOT	Human Organ Transplant Panel
RT-PCR	reverse transcription polymerase chain reaction
PBT	pathogenesis-based transcript set
FFPE	formalin-fixed paraffin-embedded
DeaKAF	deterioration of kidney allograft function
DC-GS	death-censored graft survival
DSA	donor specific antibody
KRT	kidney replacement therapy
QoL	quality of life
MP	methyloprednisolone
Tac	tacrolimus
MMF	mycophenolate mofetil
EVR	everolimus
ATG	anti-thymocyte globulin
CNI	calcineurin inhibitors
VGCV	valganciclovir
dnDSA	de novo donor specific antibody
CKTR	chronic kidney transplant rejection
NGS	next generation sequencing
iR	immune repertoire
BCR	B-cell receptor
IGH	immunoglobulin heavy chain
